# Identification of Circulating Diagnostic Biomarkers for Coronary Microvascular Disease in Postmenopausal Women Using Machine-Learning Techniques

**DOI:** 10.3390/metabo11060339

**Published:** 2021-05-25

**Authors:** Alicia Arredondo Eve, Elif Tunc, Yu-Jeh Liu, Saumya Agrawal, Huriye Erbak Yilmaz, Sadık Volkan Emren, Filiz Akyıldız Akçay, Luidmila Mainzer, Justina Žurauskienė, Zeynep Madak Erdogan

**Affiliations:** 1 Department of Food Science and Human Nutrition, Division of Nutritional Sciences, University of Illinois at Urbana-Champaign, Urbana, IL 61801, USA; aliciaa2@illinois.edu (A.A.E.); mdeliftunc@gmail.com (E.T.); yliu233@illinois.edu (Y.-J.L.); 2 Research and Training Hospital, Katip Celebi University, Izmir 35620, Turkey; huriyeerbak@hotmail.com (H.E.Y.); vemren@hotmail.com (S.V.E.); drfilizkrd@hotmail.com (F.A.A.); 3 Department of Computer Science, University of Illinois, Urbana-Champaign, Urbana, IL 61801, USA; saumyaa2@illinois.edu; 4 Izmir Biomedicine and Genome Center, Balcova, Izmir 35340, Turkey; 5 Carl R. Woese Institute for Genomic Biology, University of Illinois at Urbana-Champaign, Urbana, IL 61801, USA; lmainzer@illinois.edu (L.M.); justina@illinois.edu (J.Ž.); 6 National Center for Supercomputing Applications, University of Illinois at Urbana-Champaign, Urbana, IL 61801, USA; 7 Centre for Computational Biology, University of Birmingham, Birmingham B15 2T, UK; 8 Institute of Cancer and Genomic Sciences, University of Birmingham, Birmingham B15 2TT, UK; 9 Cancer Center at Illinois, University of Illinois, Urbana-Champaign, Urbana, IL 61801, USA; 10 Beckman Institute for Advanced Science and Technology, University of Illinois at Urbana-Champaign, Urbana, IL 61801, USA

**Keywords:** metabolic-circulating biomarker, coronary microvascular dysfunction, postmenopausal women

## Abstract

Coronary microvascular disease (CMD) is a common form of heart disease in postmenopausal women. It is not due to plaque formation but dysfunction of microvessels that feed the heart muscle. The majority of the patients do not receive a proper diagnosis, are discharged prematurely and must go back to the hospital with persistent symptoms. Because of the lack of diagnostic biomarkers, in the current study, we focused on identifying novel circulating biomarkers of CMV (cytomegalovirus) that could potentially be used for developing a diagnostic test. We hypothesized that plasma metabolite composition is different for postmenopausal women with no heart disease, CAD (coronary artery disease), or CMD. A total of 70 postmenopausal women, 26 healthy individuals, 23 individuals with CMD and 21 individuals with CAD were recruited. Their full health screening and tests were completed. Basic cardiac examination, including detailed clinical history, additional disease and prescribed drugs, were noted. Electrocardiograph, transthoracic echocardiography and laboratory analysis were also obtained. Additionally, we performed full metabolite profiling of plasma samples from these individuals using gas chromatography-mass spectrometry (GC–MS) analysis, identified and classified circulating biomarkers using machine learning approaches. Stearic acid and ornithine levels were significantly higher in postmenopausal women with CMD. In contrast, valine levels were higher for women with CAD. Our research identified potential circulating plasma biomarkers of this debilitating heart disease in postmenopausal women, which will have a clinical impact on diagnostic test design in the future.

## 1. Introduction

Cardiovascular disease (CVD) is a major public health concern. It is the main cause of morbidity among women in the US [1]. Despite similar obesity prevalence in men and women, the risk of developing cardiovascular disease is greater in obese women (64%) than in obese men (46%) [2]. Additionally, women have a higher prevalence of coronary microvascular disease (CMD). In contrast, men tend to have atheroma and epicardial endothelial dysfunction or coronary artery disease [3]. The majority of female patients having CMD are postmenopausal women, and hormone-replacement therapies (HRT) decrease CMD risk up to 30% in this population, suggesting a role for estrogens in the development and progression of CMD [4].

CMD does not involve plaque formation, which blocks blood flow in the main coronary arteries. Instead, damage to the inner walls of blood vessels manifests via spasms and decreases blood flow to the heart muscle [5]. Patients complain about chest pain, shortness of breath, fatigue, and sleep problems. The current diagnosis strategy involves invasive and expansive methods, such as CFR, IMR or PET examination, which rules out the presence of CAD. Since there are no standard and specific diagnostic tests for CMD, patients do not receive a positive diagnosis when tested for coronary artery disease. Therefore, there is a clinical need for novel ways to diagnose, treat and prevent CMD in postmenopausal women.

Our objective was to identify novel markers of CMD in postmenopausal women. In this current study, our hypothesis is that postmenopausal women with CMD will have a distinct plasma metabolite profile compared to healthy women and women with CAD. Biomarkers we identified can be utilized in the clinic to diagnose women with CMD and significantly reduce future hospitalizations, cost and time for healthcare for diverse communities.

## 2. Results

Participants of the study were postmenopausal women with similar age and body mass indexes. Patients with angina or angina equivalent underwent nonischemic testing, in particular myocardial perfusion scintigraphy. Those who had severe epicardial coronary artery disease were included in CAD groups. Those who had normal or noncritical coronary stenosis with ischemia on myocardial perfusion imaging were included in the CMD group. Baseline characteristics are provided in Table 1. There was no statistically significant difference between the group averages of LDL, triglyceride, AST, ALT, urea, creatine, Na, K, glucose, Hb, WBC, PLT and MCV values. There was a significant difference between the values of total cholesterol (*p*-value: 0.0072) and HDL (*p*-value: 0.0039). The treatment protocols of the patients were compatible with existing co-morbid diseases and cardiac risk factors. Transthoracic echocardiography (systolic dysfunction, diastolic dysfunction, valve disorder, lv hypertrophy, pulmonary hypertension) and coronary angiography results of the patients are presented in Table 2. In the CAD group, 4 patients had more than one vessel atherosclerotic heart disease, 9 patients had single-vessel stents, 3 patients had 2 vessel stents, 3 patients had 3 vessel stents. 1 patient had undergone a coronary artery bypass graft operation. Coronary angiography of 22 patients in the CMD group was normal, and 1 patient had a single-vessel noncritical plaque.

**Figure 1:**
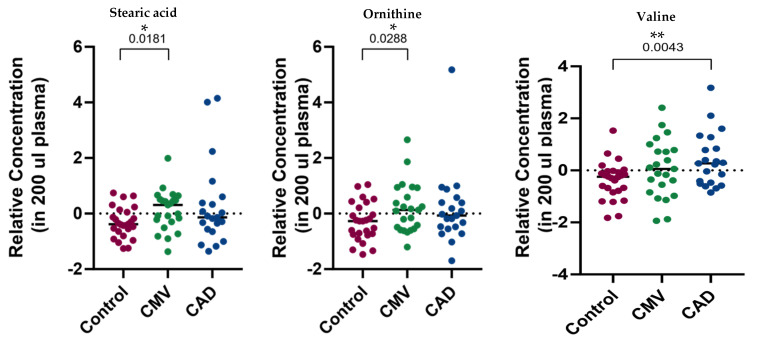
Significantly different metabolites between CMD vs. control and CAD vs. control groups, (*t*-test: * *p* < 0.01, ** *p* < 0.05).

To identify circulating biomarkers from plasma, we performed whole metabolite profiling using GC/MS analysis. The original dataset contained 175 metabolites. After data preprocessing and feature selection, we ended up with 45 metabolites. Using an unpaired t-test, we identified high stearic acid and ornithine as an indicator of CMD and valine as statistically different in the CAD group compared to the control group (Figure 1).

**Figure 2:**
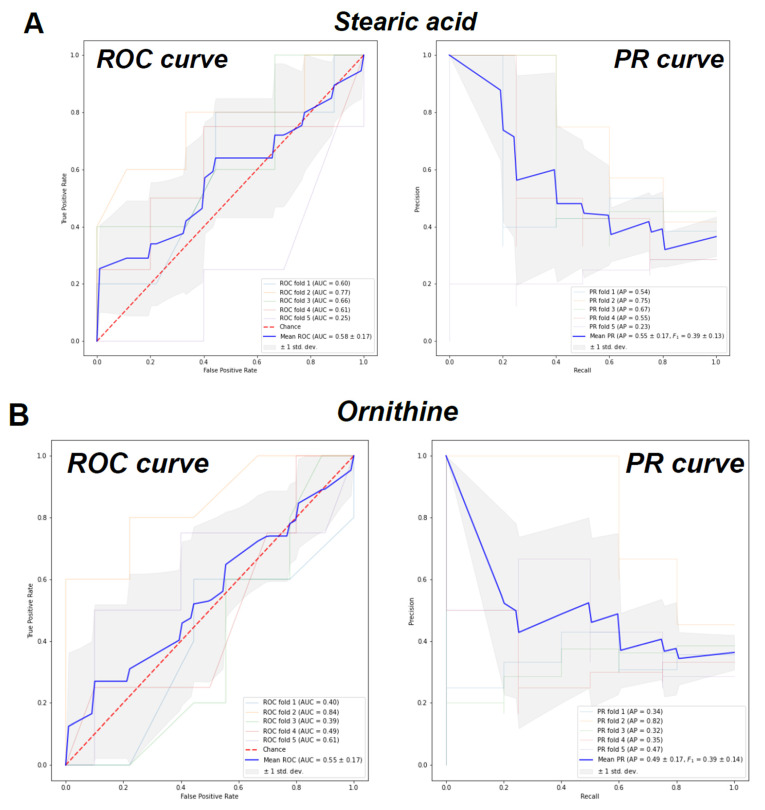

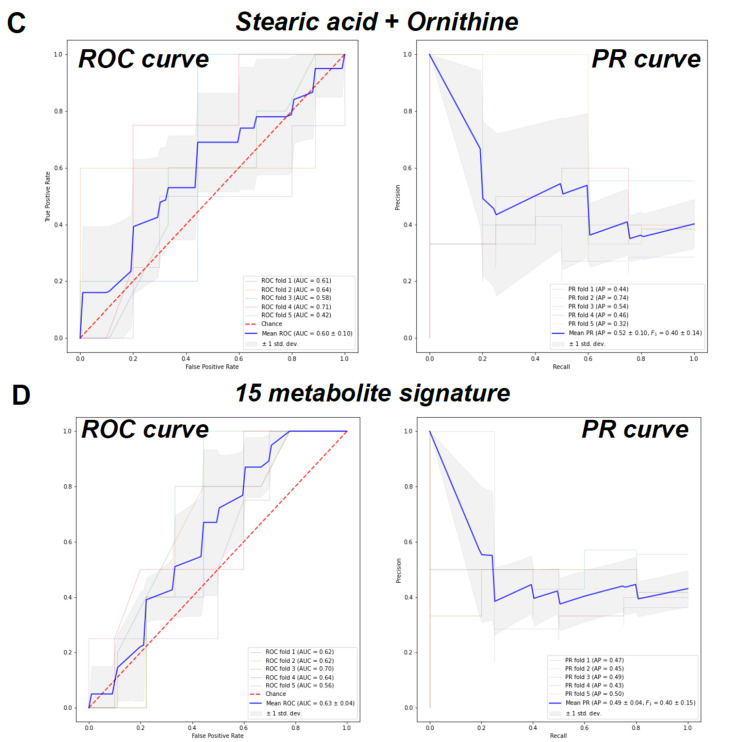
Classification performance validation using (**A**) stearic acid, (**B**) ornithine, (**C**) stearic acid + ornithine and (**D**) 15 metabolite signature.

To further develop a biomarker signature set, we used the RFECV algorithm, which iteratively computed the cross-validation score each time it eliminated a metabolite feature column (Figure S1). The classification achieved significantly better scores when more than 10 features were selected. The highest score was obtained when 15 metabolites were chosen (Figure S2). To test the classification performance of the identified signature, a binary classification was implemented using the random forest algorithm. The binary labels were “CMD” and “non-CMD”, which combined both the CAD and control group samples. We tested the classification performance of stearic acid only, ornithine only, stearic acid and ornithine together, and the 15 metabolites set. The training and testing were done using 5-fold cross-validation with a fixed random seed. The resulting performance was measured and visualized with receiver operating curves (ROC) and precision–recall (PR) curves. For the stearic acid, the mean area under the curve (AUC) was computed to be 0.58 with a standard deviation of 0.17 (Figure 2A, left panel). The mean F_1 score was computed to be 0.39 with a standard deviation of 0.13 (Figure 2A, right panel). The mean area under the curve (AUC) for ornithine was computed to be 0.55 with a standard deviation of 0.17 (Figure 2B, left panel). The mean F_1 score was computed to be 0.39 with a standard deviation of 0.14 (Figure 2B, right panel). Third, using the metabolite data from both stearic acid and ornithine, the mean area under the curve (AUC) was computed to be 0.60 with a standard deviation of 0.10 (Figure 2C, left panel). The mean F_1 score was computed to be 0.40 with a standard deviation of 0.14 (Figure 2C, right panel). Finally, using the metabolite data from the 15 metabolites selected by RFECV, the mean area under the curve (AUC) was computed to be 0.63 with a standard deviation of 0.04 (Figure 2D, left panel). The mean F_1 score was computed to be 0.40 with a standard deviation of 0.15 (Figure 2D, right panel).

## 3. Discussion

There are no specific diagnostic biomarkers for CMD. Because of the lack of specific diagnostic tests, many women who go to the emergency room with angina are discharged without a proper diagnosis, only to return to the hospital with more severe cardiac symptoms. We previously employed machine-learning techniques to identify biomarkers associated with other conditions, e.g., long-term-estrogen use [6], breast cancer risk [7], long-term broccoli consumption [8], and early predictors of liver carcinogenesis [9]. Feature selection and classification-based approaches enabled us to identify the best set of biological molecules that indicate health states with high specificity and selectivity. In the current study, we utilized plasma whole metabolite profiles and machine-learning algorithms to identify specific biomarkers of CMD. When we used classical statistical methods, which yielded stearic acid and ornithine as the metabolites that are statistically significant, they did not perform well to differentiate between CMD and non-CMD patients with high selectivity and specificity, hence lower mean AUC values. When we included 15 metabolites that were identified by the algorithm, our mean AUC increased, suggesting nonlinear methods may provide us a better set of biomarkers to develop as a diagnostic tool. However, plasma metabolites may not be ideal indicators of CMD, and current studies are underway to identify protein biomarkers, which we found to be better biomarkers for other pathological conditions before [7,10].

Long-chain acyl-carnitines, such as stearic acid, are formed from carnitine and acyl-CoAs by carnitine acyltransferases in mitochondria [11,12]. Because long-chain fatty Acids are the main energy substrates in the skeletal muscles and the heart, these tissues are considered essential contributors to the long-chain acyl-carnitine pool in plasma. The elevated levels of plasma acyl-carnitine have been linked to the progression of various diseases, including insulin resistance [13]; obesity, impaired glucose tolerance, type 2 diabetes [14,15,16]; and cardiovascular diseases [17,18,19]. In a cross-sectional study of 741 Chinese patients with T2DM, which aimed to estimate if there was an association between acyl-carnitine metabolites and CVD, investigators measured the fasting plasma levels of 25 acyl-carnitine metabolites in 288 individuals with CVD. Their analysis showed that some acyl-carnitine’s were associated with the risk of CVD in T2DM [20]. In another case-cohort study (based on PREDIMED trial) with 980 individuals (229 cases and 751 non-cases), analysis of 3 acyl-carnitine groups (short-chain, medium-chain, and long-chain) found that increased short-chain and medium-chain plasma acyl-carnitines are associated with a higher risk of CVD independent of established CVD risk factors [21]. They suggested using short-chain acyl-carnitines as a potential biomarker of future stroke risk due to its increased concentration 3–4 years before the onset of CVD [21]. Consistent with their findings suggesting no association between stearic acid and CAD, in our study, we found an association between stearic acid and CMD, but not CAD. Long-chain saturated fatty acids, such as stearic acid consumed in significant amounts in the diet, can lead to metabolic disorder of glucose and lipids [22], but also can induce vascular endothelial inflammation, which has the potential to cause vascular dysfunction [23], ultimately causing diverse cardiovascular diseases. Some studies have found that consuming a Mediterranean diet could potentially decrease the cardiovascular risks that saturated fatty acids may yield [24,25].

In CMD vasospasm, vasoconstriction in smaller arteries of the heart is a major problem. In our study, we found ornithine plasma levels to be significantly higher in postmenopausal women with CMD. Ornithine is a metabolite, which is part of the pathway for NO production and arginine catabolism. Ornithine might be due to the increased activity of Arginase I enzyme, which is expressed in coronary microvasculature and can compete with nitric oxide synthase (NOS) for the use of L-arginine as a substrate [26], which results in the generation of ornithine and urea, instead of NO and L-citrulline. Obesity caused by a chronic high-fat diet plays a role in vascular endothelial cell dysfunction because of its low-grade, chronic inflammation [27], especially in the liver, where Arginase I is upregulated, contributing to changes in arginine metabolism [28]. In many studies, it is shown that obesity increases the expression of arginase I, which alters the bioavailability of arginine [29]. Arginine can be hydrolyzed to form urea and ornithine by arginase or can form NO by nitric oxide synthase. Both pathways compete for arginine [30]. Because of the increase in Arginase I activity, there is a reduced nitric oxide production, which is a major regulator of vascular homeostasis produced by endothelial cells, which play a role in maintaining normal vascular function by modulating vascular tone, inflammation and homeostasis [31].

CMD is a disease characterized by damage to the walls and inner lining of small coronary artery blood vessels due to constrictions and spasms of the heart wall. Additionally, decreased levels of estrogen, seen in postmenopausal women, are strongly correlated to coronary microvascular disease since estrogen modulates factors that are critical for the regulation of vascular relaxation. Estrogens improve nitric oxide function, which is a potent vasodilator [5]. One of the complications of T2DM is progression to cardiovascular disease (CMD), specifically due to microvascular dysfunction [32]. Arginase is believed to be an important regulator of NO bioavailability and endothelial function by hydrolyzing L-carnitine to ornithine and urea [31]. When Arginase activity is increased, it may reduce the production of NO and impair endothelial function, causing the microvascular disease to develop. In a prospective intervention study, which included 12 healthy individuals and 12 subjects with T2DM, plasma ratios of amino acids involved in arginase and NO synthase activities were determined [33]. It was found that individuals with T2DM had significantly higher levels of plasma ornithine than control when liquid chromatography-tandem and mass spectrometry were done. In addition, T2DM patients had higher ratios of ornithine/citrulline and ornithine/arginine than the control group, indicating increased arginase activity. This study corroborates our finding, an association between increased levels of ornithine and CMD in postmenopausal women. Another case–control study analyzed plasma levels of different endogenous substrates for arginase in 298 patients, who were divided into 2 groups: Acute coronary syndrome (ACS) group, and stable angina (SAP) group [34]. Arginine, citrulline, ornithine, and methylated form of arginine c (SDMA) levels were measured using HPLC-MS/MS. Lower levels of arginine, citrulline and ornithine were found in both groups, as well as AMI (defined as the ratio of (arginine + citrulline + ornithine)/(ADMA + SMDA)). They concluded that AMI was an independent risk factor of acute coronary syndrome [34]. Findings from this study is consistent with ours, which did not find an association (no significant *p* value) between ornithine and CAD.

We identified valine, a branched-chain amino acid, to be associated with CAD. Consistent with our findings, in a double-blinded study of 73 subjects, the biochemical profile of blood plasma in subjects with CAD and normal subjects (by angiography) was analyzed by high-resolution proton NMR spectroscopy [35]. It was found that high levels of lipids, alanine, and isoleucine/leucine/valine were observed in CAD subjects when compared to the control group. Valine, just like other amino acids, such as isoleucine, aspartate, and glutamate, provides the carbon skeleton for citric acid [36] and may play a role in the myocardial adaptation and mechanism adopted for restoring the ischemic injury of the heart [35], which serves as evidence that BBAA metabolism plays a role in cardiometabolic health [37,38,39]. In a women’s health study that followed 27,041 healthy women for 18 years, isoleucine, leucine, and valine were found to be positively correlated with future CVD events [40]. Overall, our findings are consistent with these studies.

Combined with a western meat-rich diet, obesity can increase the levels of circulating BCAA (leucine, isoleucine and valine) to the point of causing non-alcoholic fatty liver disease and other related metabolic disorders by increasing the levels of free fatty acid (FFA) in plasma and in the liver, but also inhibiting the conversion of FFA into TG, causing FFA lipotoxicity in the ladder [41]. BCAA also can modulate glucose metabolism. It has been found that increase plasma concentration of BCAA is associated with the development of diabetes and insulin resistance [42].

In summary, we identified biologically relevant metabolites of microvessel function to be significantly different in postmenopausal women with CMD. Although classification performances of the identified molecules are not ideal, our studies provide proof of principle that plasma metabolite profiles can be used to develop diagnostic signatures for CMD. More future studies are underway to analyze inflammatory cytokines and other protein markers relevant to cardiovascular health. A combination of metabolomics and proteomics approaches combined with advanced machine learning techniques provides diagnostic biomarkers to be used in the clinic.

## 4. Materials and Methods

### 4.1. Study Design and Population

Studies are approved by the Izmir Katip Celebi University Interventional Clinical Studies Institutional Review Board (IRB#80). All research was carried out in compliance with the Helsinki Declaration. Donors provided broadly written consent for using their specimens in research. The consent document informed the donor that the donated specimens and medical data would be used for the general purpose of helping to determine biomarkers of CMD in postmenopausal women.

A total of 70 patients were included in this prospective observational cohort study. 23 of these patients were diagnosed with CMD (group 1), 21 patients were diagnosed with CAD (group 2), and 26 patients were defined as the control group (group 3). Inclusion criteria were as follows: having CAD-related chest pain, a positive non-invasive imaging result, and undergoing a successful coronary angiography with patients’ consent. Exclusion criteria of the study were: being male or a premenopausal female, having a contraindication against coronary angiography. Patients who refused to participate in the study were also excluded from this research.

Anamnesis, physical examination, body mass index (BMI), medications, transthoracic echocardiogram, blood pressure measurements, and routine biochemical examinations (total cholesterol (TC), high-density lipoprotein (HDL) cholesterol, low-density lipoprotein (LDL) cholesterol, triglyceride, glucose, urea, creatinine, aspartate transaminase (AST), alanine transaminase (ALT), hemoglobin (Hb), white blood cell (WBC), mean corpuscular volume (MCV), platelet (PLT) levels) were recorded (Table 1).

Coronary angiography (CAG) procedure was applied to patients who underwent non-invasive imaging methods (cardiovascular stress test, myocardial perfusion scintigraphy) and was found to be positive. CAG was performed using the Judkins technique through the femoral or radial artery. Each coronary artery was displayed in at least two different plane images. All patients who accepted the procedure were informed about the study, and their written consent was obtained. After this procedure, a 5 cc blood sample was obtained from patients who were found to have coronary artery disease or CMD as a result of coronary angiography. After obtaining the consent of patients, who were determined as the control group with similar demographic and medical characteristics, 5 cc blood was taken. Venous blood samples were obtained from the antecubital vein. 100 µL plasma samples prepared from blood samples were stored at −80 °C for processing.

### 4.2. GC/MS Validation of the Metabolites by Whole Metabolite Profiling

To detect and quantify the circulating metabolites in plasma, a gas chromatography-mass spectrometry (GC/MS) analysis was performed at UIUC Metabolomics Center as described [7,8,9,10,43]. Briefly, 50 μL of blood plasma was extracted using 1 mL of isopropanol:acetonitrile:water (3:3:2, *v/v*) at 20 °C for 5 min. After centrifugation, 0.5 mL of supernatant was dried in a SpeedVac concentrator and subsequently derivatized in two-steps: with 50 μL methoxyamine hydrochloride (Sigma-Aldrich, St Louis, MO, USA) (40 mg/mL in pyridine) for 60 min at 50 °C, then with 50 μL MSTFA + 1% TMCS (Thermo, Waltham, MA, USA) at 70 °C for 120 min, followed by a 2-h incubation at room temperature. Hentriacontanoic acid (30 μL of 1 mg/mL) was added to each sample before derivatization for use as an internal standard for normalization. Metabolite profiles were acquired using a gas-chromatography mass-spectrometry (GC–MS) system (Agilent Inc, Santa Clara, CA, USA) consisting of an Agilent 7890 gas chromatograph, an Agilent 5975 MSD and 7683 B autosampler, as previously described [44]. Briefly, gas chromatography was performed on a ZB-5MS (60 m × 0.32 mm ID and 0.25 mm film thickness) capillary column (Phenomenex, Torrance, CA, USA). The inlet and MS interface temperatures were 250 °C, and the ion source temperature was adjusted to 230 °C. An aliquot of 1 mL was injected with a split ratio of 10:1. The helium carrier gas was kept at a constant flow rate of 2.4 mL/min. The temperature program was: 5 min isothermal heating at 70 °C, followed by an oven temperature increase of 5 °C/min to °C after which a final 10 min incubation at 310 °C was performed. The mass spectrometer was operated in positive electron impact mode (EI) at 69.9 eV ionization energy at *m*/*z* 30–800 scan range. The scan range was set at least 50 *m*/*z* above the highest anticipated fragment. The minimum quality match for minor compounds was ≥80 and for other peaks ≥90. The spectra of all chromatogram peaks were evaluated using the AMDIS 2.71 (NIST, Gaithersburg, MD, USA) using a custom-built MS database (484 unique metabolites) [11]. Tentative substances were not reported. All known artificial peaks were identified and removed before data mining. To allow comparison between samples, all data were normalized to the internal standard in each chromatogram.

Differences in metabolite profiles among control, CAD and CMD were analyzed using Prism 7.0 (GraphPad software, San Diego, CA, USA, RRID:SCR_002798), after Z-scores were calculated for each metabolite. An unpaired t-test was performed to identify metabolites that are different between each group. For clinical data, a one-way-ANOVA model and chi-squared analysis were fitted to test the statistical significance of differences between different groups, followed by Tukey’s post hoc test. *p* < 0.05 was considered significant.

### 4.3. Machine Learning Analysis: Data Preprocessing, Feature Selection and Classification

The raw data consisting of 175 metabolites measured across 70 patients was normalized using min/max scaling. Data imputation was then utilized to handle missing data and get the data ready for the classification task afterward. The metabolic feature columns, which had more than 40% of data missing, were eliminated. Next, we trained an iterative imputer on the rest of the data using the IterativeImputer class inside the Python sklearn.impute module to infill missing values. We iteratively tested the trained imputer by removing the data of one metabolite feature column and imputing the data afterward. At this step, the performance of the imputer was measured for each of the metabolites by observing the R^2^ (Figure S1). We used the R^2^ measure to eliminate the metabolite column with R^2^ < 0.3 and had more than 5% of missing values across all patients. The last step of preprocessing was data standardization. We performed mean removal and variance scaling, which gave us zero mean and unit variance for each metabolite feature column. Preprocessing step reduced the feature columns to 75 metabolites, which were passed for further analysis. Feature selection was performed on the preprocessed dataset using the recursive feature elimination with cross-validation (RFECV) function inside the sklearn. Feature selection module. A random forest classifier was used as the estimator with 5-fold cv validation.

## Figures and Tables

**Table 1:** Patient characteristics. CMD = coronary microvascular disease, CAD = coronary artery disease, BMI = body mass index, COPD = chronic obstructive pulmonary disease, ACE-ARB diuretics = angiotensin-converting enzyme inhibitor, and angiotensin receptor blocker diuretics, CA = calcium channel blockers, HDL = high-density lipoprotein, LDL = low-density lipoprotein, AST = aspartate aminotransferase, ALT = alanine aminotransferase, Na⁺ = sodium, K⁺ = potassium, WBC = white blood cell, HB = hemoglobin, PLT = platelet, MCV = mean corpuscular volume.

Characteristics	Control (*n* = 26)	CMD (*n* = 23)	CAD (*n* = 21)	*p* Value ^a^
**Patient characteristics**				
Age, mean (SD), Y	62(8)	58 (7)	62 (7)	0.2057
BMI, median (IQR) ^b^	31 (30)	30 (30)	30 (29)	0.8036
Hypertension, no.(%)	17 (65)	8 (34)	11 (52)	0.101
Diabetes, no.(%)	5 (19)	6 (26)	11 (52)	0.0412
Smoking, no.(%)	2 (7)	0 (0)	1 (4)	ND
COPD or asthma, no. (%)	2 (7)	4 (17)	3 (14)	ND
HL, no. (%)	5 (19)	5 (21)	5 (23)	ND
Rheumatology, no. (%)	0 (0)	1 (4)	1 (4)	ND
Thyroid, no. (%)	4 (15)	0 (0)	3 (14)	ND
**Medication**				
Antithrombotic, no. (%)	6 (23)	14 (60)	20 (95)	<0.0001
ACE-ARB diuretics, no. (%)	14 (53)	6 (26)	13 (61)	0.0408
CA channel blockers, no. (%)	8 (30)	3 (13)	6 (28)	0.3035
Beta blocker, no. (%)	4 (15)	6 (26)	7 (33)	0.3507
Antianginal, no. (%)	0 (0)	3 (13)	11 (52)	<0.0001
Antihyperlipidemic, no. (%)	4 (15)	10 (43)	12 (57)	0.0097
**Blood test results**				
Total cholesterol, mean (median), mg/dL	237 (234)	213 (202)	192 (185)	0.0072
HDL, mean (median), mg/dL	55 (53)	49 (47)	43 (44)	0.0039
LDL, mean (median), mg/dL	150 (147)	128 (126)	113 (99)	0.0145
Triglyceride, mean (median), mg/dL	162 (146)	172 (141)	179 (151)	0.815
Glucose, mean (median), mg/dL	113 (104)	133 (114)	127 (120)	0.2305
Urea, mean (median), mg/dL	14 (13)	13 (13)	14 (13)	0.5087
Creatinine, mean (median), mg/dL	0.8 (0.8)	0.7 (0.7)	0.7 (0.7)	0.0491
AST, mean (median), U/L	19 (18)	19 (15)	18 (17)	0.7581
ALT, mean (median), U/L	18 (17)	17 (16)	20 (17)	0.5324
Na⁺, mean (median), meq/L	140 (140)	139 (139)	140 (139)	0.6461
K⁺, mean (median), meq/L	4.3 (4.3)	4.3 (4.4)	4.4 (4.5)	0.6139
WBC, mean (median), ×109/L	7 (7)	7 (7)	8 (8)	0.3099
HB, mean (median), g/dL	23 (13)	20 (13)	18 (13)	0.8631
PLT, mean (median) ×109/L	283 (270)	275 (254)	307 (303)	0.4216
MCV, mean (median), fL	87 (87)	85 (83)	83 (82)	0.3882

^a^ *p* value for 3-group comparison using one-way ANOVA multiple comparison and chi-squared analysis, ^b^ Calculated as weight in kilograms divided by height in meters squared.

**Table 2:** Characteristics of patients with CAD or CMD.

Characteristics	Control (*n* = 26)	CMD (*n* = 23)	CAD (*n* = 21)
**Transthoracic echocardiography**			
Systolic dysfunction	1	1	0
Diastolic dysfunction	15	11	13
Valve disorder	10	12	8
LV hypertrophy	4	1	2
Pulmonary hypertension	1	1	0
Coronary angiography			
Normal		22	
Atherosclerotic heart disease		1	4

## Data Availability

Data is available from authors upon request.

## Supplementary Materials

The following are available online at https://www.mdpi.com/article/10.3390/metabo11060339/s1, Figure S1: RFECV algorithm results. RFEVC iteratively computed the cross-validation score each time it eliminated a metabolite feature column, Figure S2: Relative abundance of 15 metabolites in Control, CMD and CAD groups that gave the highest ROC in when random forest algorithm was used.
