# Supplementary material for: Identification of Circulating Diagnostic Biomarkers for Coronary Microvascular Disease in Postmenopausal Women Using Machine-Learning Techniques
Source: Metabolites. 2021 May 25;11(6):339. doi: 10.3390/metabo11060339 (PMC8230313; doi:10.3390/metabo11060339)
Supplement: Supplementary file 1 [file metabolites-11-00339-s001.zip › metabolites-1196210-SI.pdf]

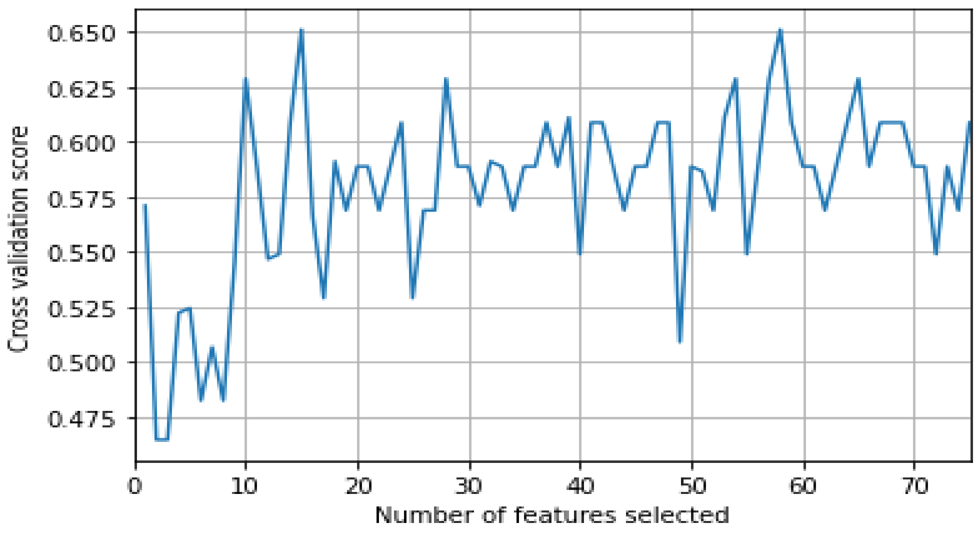

Figure S1. RFECV algorithm results. RFEVC iteratively computed the cross-validation score each time it eliminated a metabolite feature column

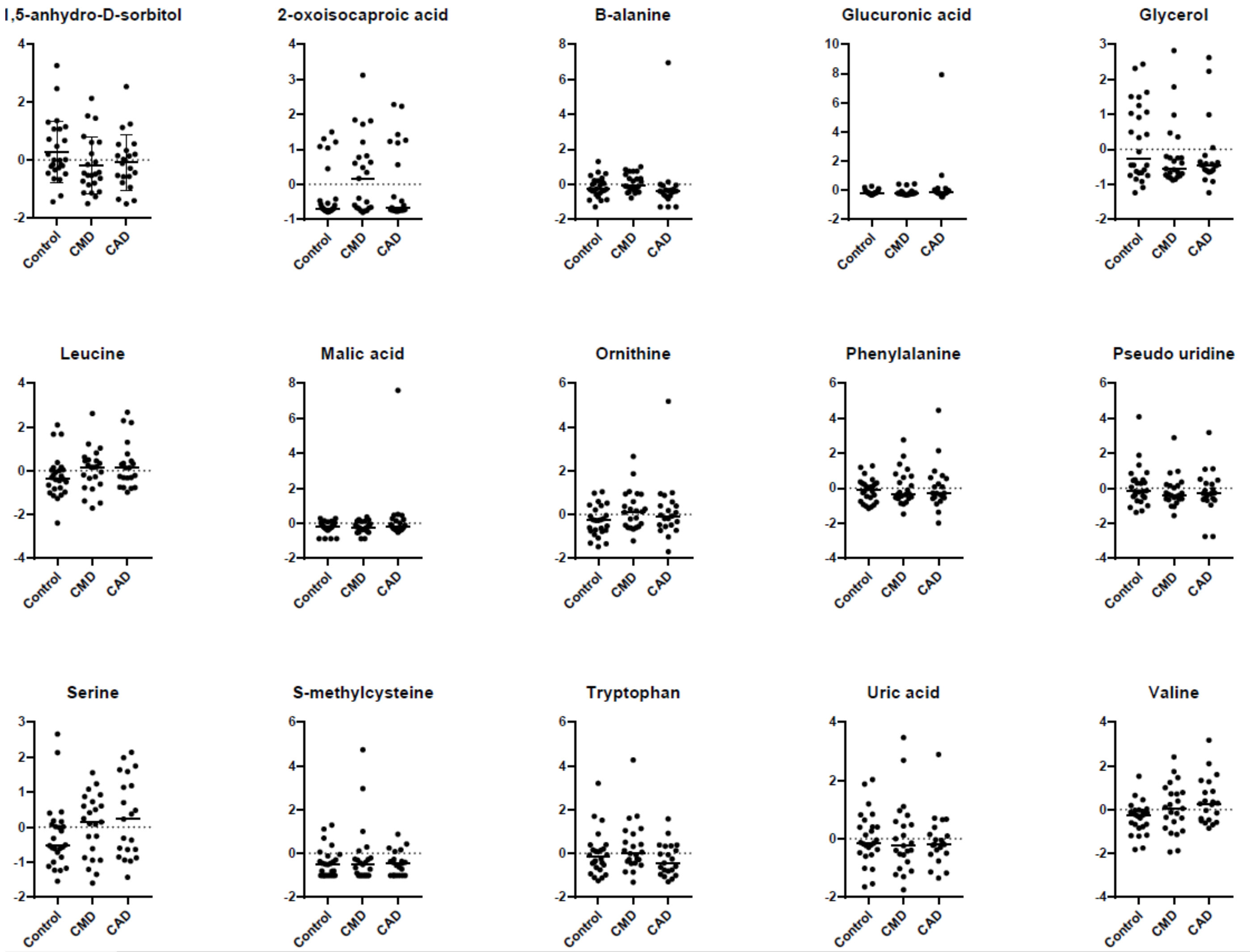

Figure S2. Relative abundance of 15 metabolites in Control, CMD and CAD groups that gave the highest ROC in when random forest algorithm was used.
